# Functional characterization of EZH2β reveals the increased complexity of EZH2 isoforms involved in the regulation of mammalian gene expression

**DOI:** 10.1186/1756-8935-6-3

**Published:** 2013-02-28

**Authors:** Adrienne Grzenda, Gwen Lomberk, Phyllis Svingen, Angela Mathison, Ezequiel Calvo, Juan Iovanna, Yuning Xiong, William Faubion, Raul Urrutia

**Affiliations:** 1 Laboratory of Epigenetics and Chromatin Dynamics, Mayo Clinic, Rochester, MN, 55905, USA; 2 Molecular Endocrinology and Oncology Research Center, CHUL Research Center, Quebec, Canada; 3 INSERM U.624, Stress Cellulaire, 163 Avenue de Luminy, Case 915, Parc Scientifique et Technologique de Luminy, Marseille Cedex 9, 13288, France; 4 Translational Epigenomics Program, Center for Individualized Medicine (CIM), Mayo Clinic, Rochester, MN, 55905, USA; 5 Departments of Medicine, Physiology and Biochemistry, Mayo Clinic, Rochester, MN, 55905, USA

**Keywords:** Chromatin, Enhancer of zeste homologue 2, Epigenetics, EZH2, Histone methyltransferase, Polycomb, Polycomb repressive complex 2, PRC2

## Abstract

**Background:**

Histone methyltransferase enhancer of zeste homologue 2 (EZH2) forms an obligate repressive complex with suppressor of zeste 12 and embryonic ectoderm development, which is thought, along with EZH1, to be primarily responsible for mediating Polycomb-dependent gene silencing. Polycomb-mediated repression influences gene expression across the entire gamut of biological processes, including development, differentiation and cellular proliferation. Deregulation of EZH2 expression is implicated in numerous complex human diseases. To date, most EZH2-mediated function has been primarily ascribed to a single protein product of the *EZH2* locus.

**Results:**

We report that the *EZH2* locus undergoes alternative splicing to yield at least two structurally and functionally distinct EZH2 methyltransferases. The longest protein encoded by this locus is the conventional enzyme, which we refer to as EZH2α, whereas EZH2β, characterized here, represents a novel isoform. We find that EZH2β localizes to the cell nucleus, complexes with embryonic ectoderm development and suppressor of zeste 12, trimethylates histone 3 at lysine 27, and mediates silencing of target promoters. At the cell biological level, we find that increased EZH2β induces cell proliferation, demonstrating that this protein is functional in the regulation of processes previously attributed to EZH2α. Biochemically, through the use of genome-wide expression profiling, we demonstrate that EZH2β governs a pattern of gene repression that is often ontologically redundant from that of EZH2α, but also divergent for a wide variety of specific target genes.

**Conclusions:**

Combined, these results demonstrate that an expanded repertoire of EZH2 writers can modulate histone code instruction during histone 3 lysine 27-mediated gene silencing. These data support the notion that the regulation of EZH2-mediated gene silencing is more complex than previously anticipated and should guide the design and interpretation of future studies aimed at understanding the biochemical and biological roles of this important family of epigenomic regulators.

## Background

The currently accepted hierarchical model of Polycomb-mediated gene repression begins with the trimethylation of histone 3 at lysine 27 (H3-K27me3) through the action of Polycomb repressive complex (PRC) 2, a multi-subunit complex. The H3-K27me3 mark subsequently recruits PRC1, leading to the propagation of the repressed state through a variety of mechanisms, including chromatin compaction and recruitment of other chromatin-remodeling enzymes, such as DNA methyltransferases [1]. However, it must be noted that alternative pathways for PRC1 recruitment, independent of inscription of the H3-K27me3 mark, have also been observed [2]. The enzymatic engine of PRC2 is enhancer of zeste homologue 2 (EZH2), which possesses the evolutionarily conserved SET domain that confers the complex with its histone methyltransferase (HMT) activity [3,4]. The catalytic function of EZH2 is strictly dependent on the presence of both WD40-repeat protein embryonic ectoderm development (EED) and zinc finger protein suppressor of zeste 12 (SUZ12), which serve to link and stabilize the enzyme to its histone substrate [5-9]. A number of other proteins, such as retinoblastoma binding proteins 4 and 7 (RBBP4/7), Adipocyte enhancer-binding protein 2 and PHD finger protein 1 enhance the enzymatic function of the complex [5,10-12]. Thus, within these complexes, EZH2 proteins serve as the key histone code writers of the H3-K27me3 mark that leads to long-term epigenetic gene silencing.

PRC2 proteins are extremely well conserved from plants to humans, indicating the fundamental importance of this epigenetic mechanism to organism development and survival [13]. Interestingly, several alternative PRC2 complexes have been identified. EED undergoes alternative translation to yield four protein products. PRC2 complex was defined as a complex between EZH2, SUZ12, RBBP4/7 and the longest isoform of EED, EED1. However, a second complex, PRC3, was found with the substitution of the shortest isoforms of EED, EED3/4, that was capable of H3-K27 methylation [14]. Another alternative complex, PRC4, was found with the substitution of EED2, an isoform normally undetectable in differentiated cells but prevalent in embryonic stem cells and transformed tissues [15]. EZH1, a homologue of EZH2 encoded at a separate locus, is also capable of H3-K27 trimethylation and transcriptional silencing. EZH1 forms a non-canonical PRC2 complex with EED and SUZ12. However, current evidence supports the idea that PRC2-EZH1-mediated H3-K27 trimethylation is less widespread than for PRC2-EZH2. PRC2-EZH1, for instance, has been shown to repress transcription through chromatin compaction in the absence of methyltransferase co-factor S-adenosyl methionine [10]. Together, these data suggest a more intricate regulation of H3-K37me3 deposition than previously anticipated.

The multiplicity of downstream biological functions mediated by EZH2 points to a pervasiveness of Polycomb-mediated repression well beyond development. Roles for EZH2 have been identified in cell cycle, cellular differentiation and pluripotency, among many others [16]. Pathologically, EZH2 has been implicated in the neoplastic transformation of a number of cell types, including for many solid tumors and hematopoietic malignancies. Levels of EZH2 strongly associate with the severity of malignant progression and poor prognosis in breast and prostate cancer [17,18]. The medical relevance of this observation is congruent with the functions for EZH2 as revealed by experimental methods. For instance, EZH2 overexpression promotes cellular proliferation [19-24], migration [25-27], angiogenesis [28] and survival [29,30]. Thus, both basic and translational investigations have established a solid role for EZH2 and its partners as an epigenetic system involved in oncogenesis (epigenetic oncogenes), for which detailed mechanisms underlying their function have become an area of intensive investigation.

The current study increases our knowledge on the complexity of EZH2-mediated processes by providing biochemical evidence revealing extended isoform diversity within EZH2 proteins that function in mammalian cells. Indeed, our molecular and functional data indicate that the *EZH2* locus encodes a novel isoform, EZH2β. This isoform localizes to the cell nucleus, complexes with EED and SUZ12, and binds to promoters where it increases H3-K27me3 levels, all properties in common with EZH2α protein. Importantly, however, EZH2β participates in the regulation of gene expression with a pattern that is not only shared but also distinct from that regulated by EZH2α, pointing to both redundancy and specialization within members of this HMT family of proteins. Combined, these results reveal that the regulation of H3-K27 methylation is more complex than previously anticipated and expands our knowledge of how cells generate and use different histone code writers to achieve distinct biochemical and biological functions. This new knowledge must be taken into consideration in the design and interpretation of studies on gene expression, distinct cell functions, single target gene promoters or genome-wide epigenomics, as it reveals for the first time the need for isoform-specific tools to dissect Polycomb functions.

## Results

### Identification of EZH2β reveals the existence of an expanded repertoire of EZH2 isoforms widely expressed in human tissues

**Figure 1:**
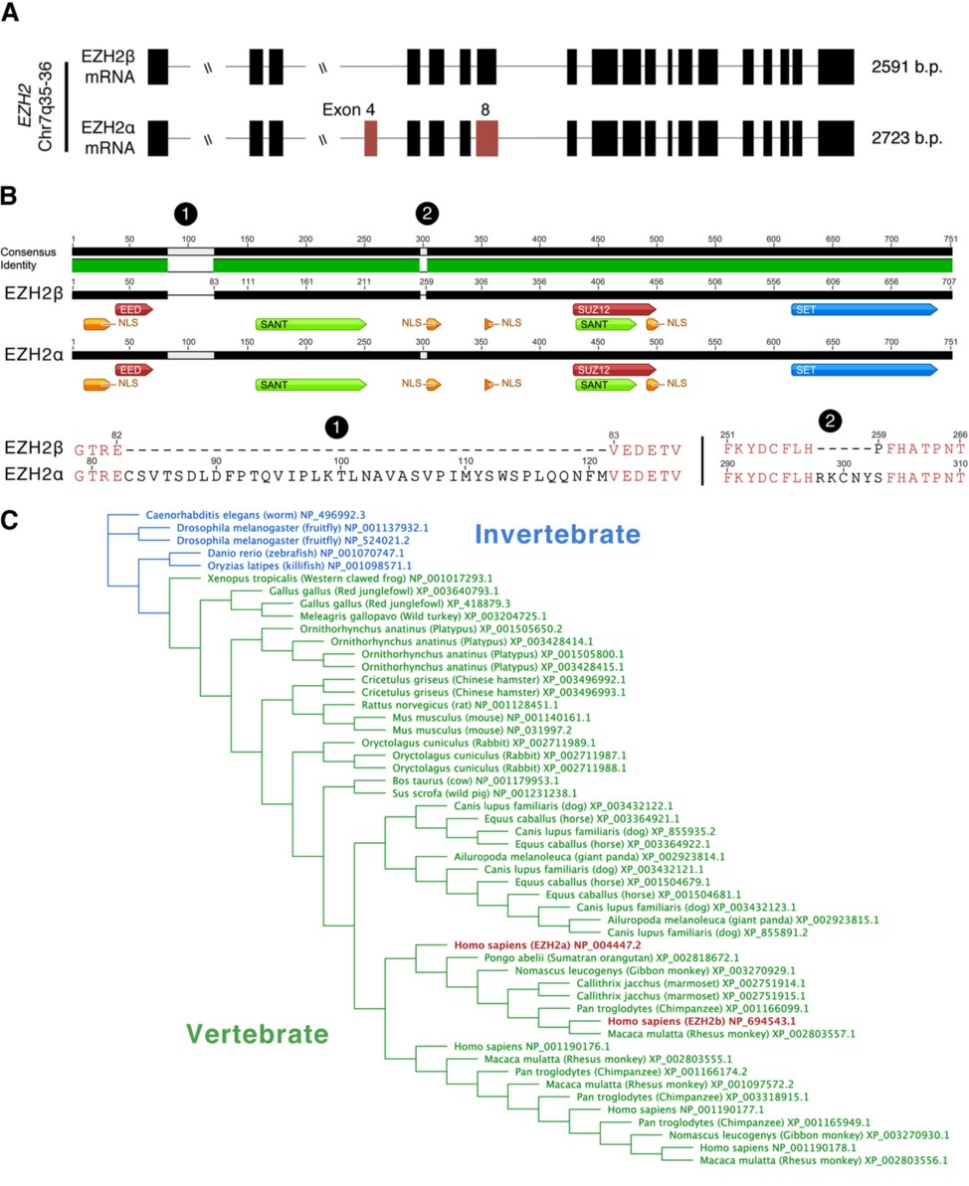
**The *EZH2 *locus yields two major transcriptional variants: EZH2α and EZH2β.** (**A**) Comparative analysis of the structure of EZH2α and EZH2β transcript variants where sites of alternative splicing events are highlighted in red on the reference isoform. Details of splicing events are described in Table 1. (**B**) Protein structure differences between EZH2α and EZH2β proteins shows the conservation of functional domains and binding sites of enzymatic co-factors, represented by different colors and labeled accordingly. Labels 1 and 2 indicate the locations of deletions in EZH2β compared with EZH2α. A comparison of the amino acid sequence highlighting the amino acid differences between the two proteins is also presented. (**C**) Evolutionary dendrogram of invertebrate (blue) and vertebrate (green) EZH2 isoforms. Nodes are spaced according to evolutionary distance. Human EZH2α and EZH2β are highlighted in red. Combined, these results reveal the potential of EZH2 to generate various isoforms through alternative splicing mechanisms as well as highlight the conservation of EZH2 isoforms throughout evolution. Bp: base pair; EZH2: enhancer of zeste homologue 2.

The current study initiated from investigations on the role of the *EZH2* locus in the proliferative response, as previous reports implicated overexpression of this HMT during neoplastic transformation in a variety of cancers [31]. Initial western blot analyses in pancreatic cancer cells revealed the presence of multiple EZH2-positive bands (Additional file 1: Figure S1). To date, over 30 different EZH2 mRNAs have been validated by high-throughput genomic sequencing efforts. One of the proteins generated from this locus, EZH2α, encoded by 20 exons, is the HMT classically associated with the function of the PRC2 complex (Figure 1A and Table 1). EZH2β, a novel isoform that the current study functionally characterizes in better detail, skips exon 4 of EZH2α and utilizes an alternative 5^′^ splice donor on EZH2α exon 8/EZH2β exon 7. At the protein sequence level, EZH2α and EZH2β differ by 44 amino acids, measuring 751 and 707 amino acids, respectively (Figure 1B). A highly similar third splice variant encoding five less amino acids than EZH2α has also been cited as EZH2. Structural comparison of these closely related variants does not reveal any noticeable differences that would suggest differing function and thereby have been considered interchangeable in the literature.

**Table 1:** Comparison of EZH2α and EZH2β transcripts yielded by alternative splicing of EZH2 locus transcripts

**EZH2α**					**EZH2β**				
**Exon**	**Exon** **size**	**5**^′^**donor**	**3**^′^**acceptor**	**Intron** **size**	**Exon**	**Exon** **size**	**5**^′^**donor**	**3**^′^**acceptor**	**Intron** **size**
1	186	ACGAAGgtaacgc	cttttagAATAAT	36,858	1	186	ACGAAGgtaacgc	cttttagAATAAT	36,858
2	124	GTAAAGgtataat	ttaaagAGTATG	583	2	124	GTAAAGgtataat	ttaaagAGTATG	583
3	129	AGGGAGgttggtt	gttttagTGTTCG	13,719	3	129	AGGGAGgttggtt	ttttagGTGGAA	16,621
4	117	TTTATGgtatgta	ttttagGTGGAA	2,785	4	121	ATAGAGgtgagcc	gtttcagAATGTG	847
5	121	ATAGAGgtgagcc	gtttcagAATGTG	847	5	141	GAGATGgtatgcc	tgtttagATAAAG	1,473
6	141	GAGATGgtatgcc	tgtttagATAAAG	1,473	6	103	GGAAAAgtaagaa	atgtcagATATAA	531
7	103	GGAAAAgtaagaa	atgtcagATATAA	531	7	164	TACATCgtaagt	tttgcagCTTTTC	6,781
8	179	ATTATTgtacgtt	tttgcagCTTTTC	6,766	8	92	CATTTGgtaagac	ttcgtagGAGGGA	1,478
9	92	CATTTGgtaagac	ttcgtagGAGGGA	1,478	9	241	CCTCTGgtaagac	tttgtagAAGCAA	485
10	241	CCTCTGgtaagac	tttgtagAAGCAA	485	10	170	AGACAGgtaaga	ttgtcagGTGTAT	443
11	170	AGACAGgtaaga	ttgtcagGTGTAT	443	11	95	ACACCGgtgagtc	tttgcagGTTGTG	1,137
12	95	ACACCGgtgagtc	tttgcagGTTGTG	1,137	12	41	AAAAGGgttagca	tactcagACGGCT	466
13	41	AAAAGGgttagca	tactcagACGGCT	466	13	126	CAGAGTgtaagta	tctgaagGTCAAA	776
14	126	CAGAGTgtaagta	tctgaagGTCAAA	776	14	179	AAAAAGgtgagca	tctctagCATCTA	2,238
15	179	AAAAAGgtgagca	tctctagCATCTA	2,238	15	96	GGAGAGgtaaggc	tttttagATTATT	1,210
16	96	GGAGAGgtaaggc	tttttagATTATT	1,210	16	82	ACAATGgtatgtt	cttttagATTTTG	942
17	82	ACAATGgtatgtt	cttttagATTTTG	942	17	81	CAAAAGgtaggta	tttgcagTTATGA	154
18	81	CAAAAGgtaggta	tttgcagTTATGA	154	18	85	TTACAGgttggta	gtttcagATACAG	1,364
19	85	TTACAGgttggta	gtttcagATACAG	1,364	19	335	TTGAATCatctctc	ND	
20	335	TTGAATCatctctc	ND						

Deposited cDNA sequences for EZH2α (NM_004456.4) and EZH2β (NM_152998.2) were aligned against the most recently published human genome sequence (Feb. 2009 GRCh37/hg19) to determine splice donors, splice acceptors, and exon and intron sizes using the University of California, Santa Cruz BLAT tool. ND: not determined.

For the purposes of our manuscript, we have selected the longest transcriptional variant (751 amino acids) as the reference sequence to provide a numbering framework for our bioinformatics. Compared with this reference EZH2α sequence, the shorter EZH2β displays conservation of several domains, namely the nuclear localization signals, SANT (DNA-binding domains) and the SET domain. Together, the conservation of these domains should confer this protein with the ability to localize to the nucleus and function as an HMT (Figure 1B). Notably, the regions that have previously been described to interact with the obligate EZH2α co-factors SUZ12 and EED are also preserved in EZH2β [7,32], suggesting that different EZH2 gene products complex with molecules significant to its major biochemical function. Additionally, comparison of all currently available genome sequences generated by world-wide sequencing efforts revealed that alternative splicing of the *EZH2* locus is conserved from invertebrates to vertebrates. The number of predicted EZH2 orthologs within each of the surveyed species suggests multiple expansion and reduction events may have occurred during the evolution of the protein as evolutionary distance increases from invertebrates to higher-order mammals (Figure 1C). We find that the EZH2α and EZH2β are predicted to be greater than 99% conserved in higher-order mammals (Table 2), suggesting that these two proteins may account for a large number of the functions evolutionarily selected for the *EZH2* locus.

**Table 2:** Conservation of the EZH2α and EZH2β isoforms across species

**RefSeq**	**Species**	**Common name**	**EZH2α/NP_004447.2** **% identity**	**EZH2β/NP_694543.1** **% identity**
NP_004447.2	*Homo sapiens*	Human	100.0	94.0
NP_694543.1	*Homo sapiens*	Human	94.0	100.0
XP_002923814.1	*Ailuropoda melanoleuca*	Giant panda	98.7	92.7
XP_002923815.1	*Ailuropoda melanoleuca*	Giant panda	92.6	98.3
NP_001179953.1	*Bos taurus*	Cow	97.6	91.8
NP_496992.3	*Caenorhabditis elegans*	Worm	23.8	23.4
XP_003496992.1	*Cricetulus griseus*	Chinese hamster	96.6	92.3
XP_003496993.1	*Cricetulus griseus*	Chinese hamster	96.2	90.3
XP_002751914.1	*Callithrix jacchus*	Marmoset	97.9	93.5
XP_002751915.1	*Callithrix jacchus*	Marmoset	92.6	98.3
XP_003432121.1	*Canis lupus familiaris*	Dog	97.9	93.5
XP_003432122.1	*Canis lupus familiaris*	Dog	96.7	92.3
XP_003432123.1	*Canis lupus familiaris*	Dog	92.6	98.3
XP_855891.2	*Canis lupus familiaris*	Dog	86.9	92.3
XP_855935.2	*Canis lupus familiaris*	Dog	91.0	86.5
NP_001137932.1	*Drosophila melanogaster*	Fruit fly	53.6	51.9
NP_524021.2	*Drosophila melanogaster*	Fruit fly	53.6	51.9
NP_001070747.1	*Danio rerio*	Zebrafish	84.0	80.6
XP_001504679.1	*Equus caballus*	Horse	98.7	94.2
XP_001504681.1	*Equus caballus*	Horse	93.5	99.4
XP_003364921.1	*Equus caballus*	Horse	97.5	93.0
XP_003364922.1	*Equus caballus*	Horse	91.7	87.3
XP_003640793.1	*Gallus gallus*	Red jungle fowl	91.2	97.0
XP_418879.3	*Gallus gallus*	Red jungle fowl	96.1	92.0
XP_001097572.2	*Macaca mulatta*	Rhesus monkey	98.0	93.6
XP_002803555.1	*Macaca mulatta*	Rhesus monkey	99.2	94.8
XP_002803556.1	*Macaca mulatta*	Rhesus monkey	92.3	87.8
XP_002803557.1	*Macaca mulatta*	Rhesus monkey	94.0	100.0
XP_003204725.1	*Meleagris gallopavo*	Wild turkey	91.0	86.8
NP_001140161.1	*Mus musculus*	Mouse	97.1	91.3
NP_031997.2	*Mus musculus*	Mouse	97.6	93.3
XP_003270929.1	*Nomascus leucogenys*	Gibbon monkey	99.2	94.8
XP_003270930.1	*Nomascus leucogenys*	Gibbon monkey	92.3	87.8
XP_001505650.2	*Ornithorhynchus anatinus*	Platypus	90.4	86.1
XP_001505800.1	*Ornithorhynchus anatinus*	Platypus	92.3	98.2
XP_003428414.1	*Ornithorhynchus anatinus*	Platypus	97.3	93.0
XP_003428415.1	*Ornithorhynchus anatinus*	Platypus	92.3	98.2
XP_002711987.1	*Oryctolagus cuniculus*	Rabbit	98.3	93.8
XP_002711988.1	*Oryctolagus cuniculus*	Rabbit	93.1	99.0
XP_002711989.1	*Oryctolagus cuniculus*	Rabbit	97.9	91.9
NP_001098571.1	*Oryzias latipes*	Killifish	82.0	78.6
XP_002818672.1	*Pongo abelii*	Sumatran orangutan	95.5	90.7
XP_001165949.1	*Pan troglodytes*	Chimpanzee	92.3	87.8
XP_001166099.1	*Pan troglodytes*	Chimpanzee	94.0	100.0
XP_001166174.2	*Pan troglodytes*	Chimpanzee	99.2	94.8
XP_003318915.1	*Pan troglodytes*	Chimpanzee	98.0	93.6
NP_001128451.1	*Rattus norvegicus*	Rat	97.5	93.2
NP_001231238.1	*Sus scrofa*	Wild pig	96.7	92.2
NP_001017293.1	*Xenopus tropicalis*	Western clawed frog	93.2	89.0

Human EZH2α and EZH2β were aligned against all predicted EZH2 isoforms across species to determine the degree of conservation. The highest scoring correlations for each species and the two isoforms are indicated. Global alignment with free ends gap was performed using the Geneious alignment tool with BLOSUM62 matrix constrained by an open gap penalty of 12 and gap extension penalty of 3.

**Figure 2:**
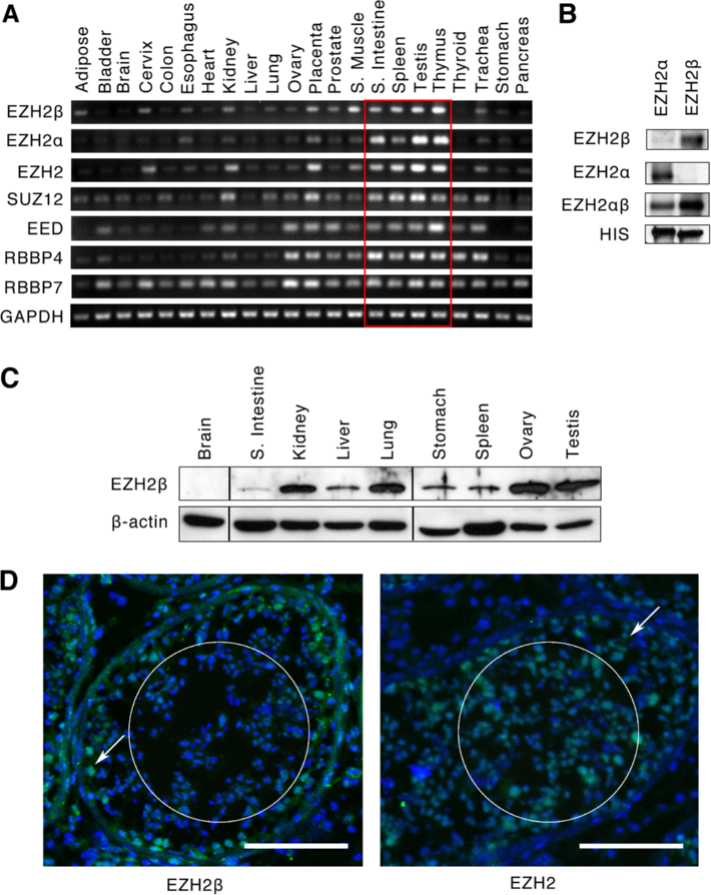
**EZH2β is expressed in a variety of adult human tissues.** (**A**) Analysis of EZH2β, EZH2α, as well as associated complex co-factors SUZ12, EED, RBBP4 and RBBP7 transcripts in 22 human tissues. RT-PCR was performed with primers designed to differentiate the two EZH2 isoforms. Analysis of glyceraldehyde-3-phosphate dehydrogenase was used as amplification control. Red box indicates tissues that possess highly proliferative capacity and preferential co-expression of all PRC2 complex transcripts, including EZH2β. (**B**) Specificity of EZH2α and EZH2β antibodies. Isoform-specific antibodies were tested using western blot of untransfected pancreatic cancer cell lines (Additional file 1: Figure S1) and also shown here in cells transfected with histidine (HIS) epitope-tagged EZH2α, EZH2β or both isoforms (EZH2αβ). Labeling of the HIS-tag was used as loading control. (**C**) Tissue distribution of EZH2β at the protein level where the presence of EZH2β was determined by western blot analysis of human tissues with the EZH2β-specific antibody. β-actin was used as loading control. (**D**) Fluorescent immunohistochemistry of samples of frozen human testis was performed by laser confocal microscopy, in sections labeled for EZH2β (left) and total EZH2 (right). The white circle encompasses primary and secondary spermatocytes, whereas primary spermatogonia are immediately adjacent to the basal lamina (white arrow). Nuclei are counterstained with Hoechst. Images were obtained at 10× magnification. White scale bar represents 100 μm. Together, these results validate that the two major isoforms generated by the human EZH2 locus, namely EZH2α and EZH2β, are translated into proteins that can be detected not only in cell lysates but also in whole tissues. EED: embryonic ectoderm development; EZH2: enhancer of zeste homologue 2; GADPH: glyceraldehyde-3-phosphate dehydrogenase; HIS: histidine; RBBP: retinoblastoma binding protein; SUZ12: suppressor of zeste 12.

Using a panel of 22 different tissues, we demonstrated that these two transcripts share a similar expression profile in most human organs. Additional comparative analyses of the expression pattern for other Polycomb-related co-factors demonstrated that tissues expressing high levels of EZH2α or EZH2β transcripts display a concordant enrichment of transcripts encoding other PRC2 complex proteins, ones previously described to be exclusive partners of the conventional enzyme, EZH2α (Figure 2A). More importantly, we validated the existence of EZH2β at the protein level using an affinity-purified antibody specifically generated against this novel isoform (Figure 2B). The electrophoretic mobility of these two isoforms as resolved by SDS-PAGE is in agreement with the molecular weights predicted from the respective amino acid compositions (EZH2α: 86.03 kDa; EZH2β: 81.05 kDa). Experiments with these specific antibodies confirmed that the EZH2β transcript is readily translated into a protein that has the potential to function as a novel member of the PRC2 complex (Figure 2C). Furthermore, the pattern of expression of EZH2β as defined by western blot was highly concordant with that predicted by PCR-based transcript analysis with a marked enrichment in testes and ovary and absence from brain. For comparison, we generated an antibody against EZH2α that, similar to all commercially available tools, primarily detects this protein but has the potential to cross-react with other EZH2 products. Our results demonstrate that our antibodies readily recognize both EZH2α and EZH2β proteins with high specificity (Figure 2B). To our surprise, we did not observe additional bands besides those corresponding to EZH2α and EZH2β, suggesting that, in spite of the large splicing potential of EZH2, these two proteins are the most readily detected products generated from this locus in human cells. Next, given the high degree of expression shown for EZH2β by our transcriptional and proteomic screens, we performed immunohistochemistry against this protein in human testis (Figure 2D), where cell fate determination relies on two pools with different proliferative mechanisms, namely meiosis and mitosis. Interestingly, the EZH2β-specific antibody revealed its preferential expression in the primary spermatogonia nuclei (white arrow, Figure 2D, left), a subpopulation of cells that are concentrated along the basal lamina and actively undergoing mitosis. Staining for EZH2β was absent in the nuclei of primary and secondary spermatocytes that are located more centrally in the tubules and divide by meiosis (white circle, Figure 2D, left). This difference in labeling hints at differences in the function of EZH2β versus the total EZH2 pool. Comparatively, the use of a well-characterized EZH2α antibody revealed immunoreactivity across all cellular types within seminiferous tubules, including in the nuclei of spermatogonia as well as primary and secondary spermatocytes (white circle, Figure 2D, right).

Our combined results demonstrate that the *EZH2* locus gives rise to various isoforms, confirms the existence of two major isoforms (EZH2α and EZH2β) at both the mRNA and protein levels, and shows their localization in tissue by immunohistochemistry. These experimental datasets encouraged us to subsequently perform functional studies to test whether these different EZH2 isoforms play redundant or overlapping functions in human cells by analyzing their cellular localization, co-factor binding, and behavior as H3-K27 writers during gene silencing on an isolated gene promoter, as well as their genome-wide effects on gene expression. We also sought to gain insight into their biological function by analyzing their effect on cell proliferation, one of the best-characterized functions attributed to the EZH2 locus in normal morphogenesis and cancer.

### EZH2β localizes to the nucleus and interacts with SUZ12 and EED

**Figure 3:**
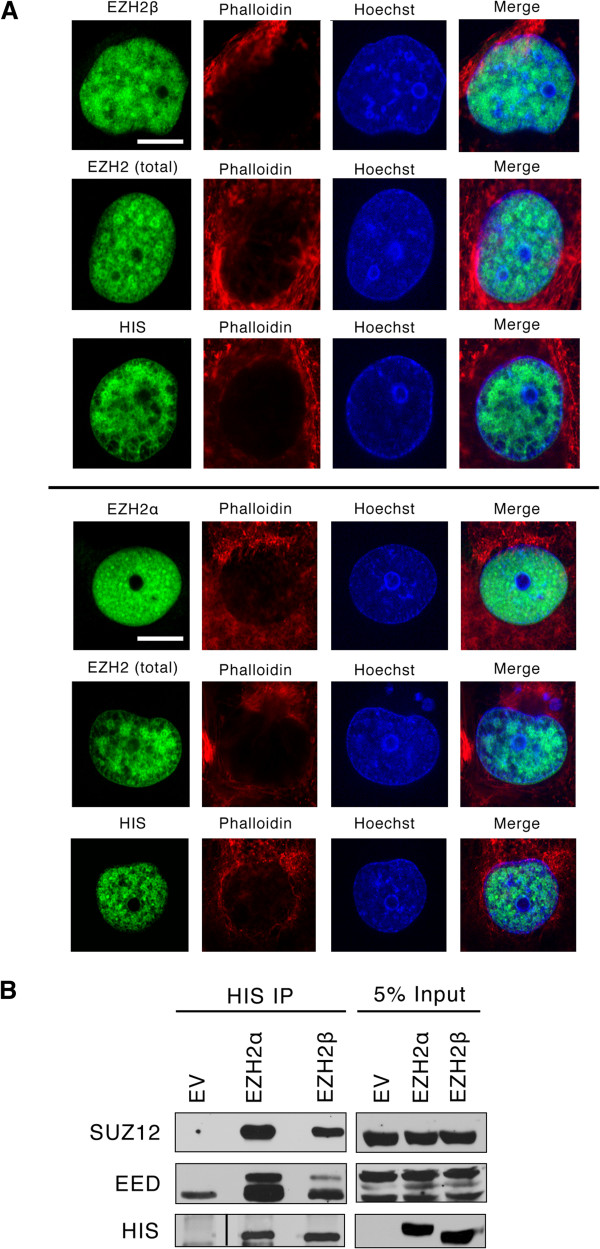
**EZH2β is localized to the nucleus and partners with SUZ12 and EED.** (**A**) Subcellular localization of EZH2β. Antibodies against total EZH2, EZH2α or EZH2β were used to determine EZH2α or EZH2β localization by immunofluorescence of epithelial cells transduced with HIS/EZH2β or HIS/EZH2α. Labeling of the HIS-tag was performed to both confirm localization and control for expression. Nuclei are counterstained with Hoechst and cytoskeletal components labeled with phalloidin red. Images were taken at 100× magnification. White scale bar represents 10 μm. (**B**) EZH2β interaction with SUZ12 and EED. Immunoprecipitation from whole cell extracts harvested from epithelial cells transduced with HIS/EZH2α or HIS/EZH2β using an antibody against the HIS-tag were probed with antibodies against SUZ12 and EED. Five percent inputs of whole cell lysates were included as control of transduction and expression. These results demonstrate that EZH2β localizes to the nucleus and interact with PRC2 targets, which are two key features expected of a functional EZH2 isoform. EED: embryonic ectoderm development; HIS: histidine; EZH2: enhancer of zeste homologue 2; EV: empty vector; immunoprecipitation; SUZ12: suppressor of zeste 12.

EZH2 has been historically considered an exclusively nuclear protein, although previous studies have described EZH2 immunoreactivity in the cytoplasm of cancer cells [31]. This knowledge prompted us to better define the cell compartment where the new EZH2 isoform functions using confocal microscopy on isolated epithelial cells. We found that although EZH2β as well as EZH2α localize to the cell nucleus (Figure 3A), neither of our antibodies noticeably labeled the cytoplasm. Thus, although it remains possible that alternative splicing can contribute to the generation of a cytoplasmic form of EZH2, we found no evidence for this localization with EZH2β under the conditions studied.

We also performed co-immunoprecipitation experiments to define whether EZH2β interacts with other members of the PRC2 complex. We found that this protein is capable of complexing with SUZ12 and EED (Figure 3B), previously described as obligate co-factors of conventional EZH2 methyltransferase function, although the interaction between SUZ12 and EZH2β is seemingly weaker than that between SUZ12 and EZH2α. Thus, the sequences used by various EZH2 proteins for complexing to its enzymatic partners are not only conserved but also functional. Together, the results of these biochemical studies demonstrate that EZH2β shares with EZH2α the ability to localize to the cell nucleus and complex with SUZ12 and EED, suggesting that both proteins display mechanistic properties that are expected for them to participate in the regulation of gene repression, an idea which was tested at higher stringency in subsequent experiments.

### EZH2β mediates H3-K27me3-associated gene silencing on promoters for homeodomain-containing proteins in genomically integrated reporter systems and isolated murine T cells

The histone code hypothesis represents a useful paradigm for understanding how histone marks deposited by writer proteins (for example, HMTs) recruit mark readers to gene promoters, which triggers the transition between permissive and non-permissive chromatin that ultimately regulates gene transcription. Currently, the conventional EZH2 protein (EZH2α) is the most characterized writer of the H3-K27me3 mark in organisms ranging from flies to humans. For instance, via this mechanism, EZH2-containing PRCs have an evolutionarily conserved role in regulating the expression of entire families of transcriptional regulators, such as those for homeobox, Sry-related high-mobility-group box and forkhead box (FOX) families [33]. The promoter of the human *forkhead* homologue, *FOXP3*, has recently been described by our group as a PRC2-regulated gene containing one of the few identified mammalian Polycomb response elements [34]. Therefore, we used the *FOXP3* promoter as a model for assaying the transcriptional regulatory function of EZH2β. This system allowed us to test the hypothesis that, similar to EZH2α, EZH2β fulfills the criteria as a writer of the H3-K27me3 known to precipitate gene silencing. Because EZH2 function associates with long-term silencing, instead of using the typical episomally based reporter systems, we used an integrated luciferase gene system in which the *FOXP3* promoter was cloned after the cytomegalovirus (CMV) promoter (JFOXP3-FLP). This design allowed us to measure the dominant effects of EZH2-mediated silencing effects over the robust activation provided CMV promoter in a highly sensitive integrated system.

**Figure 4:**
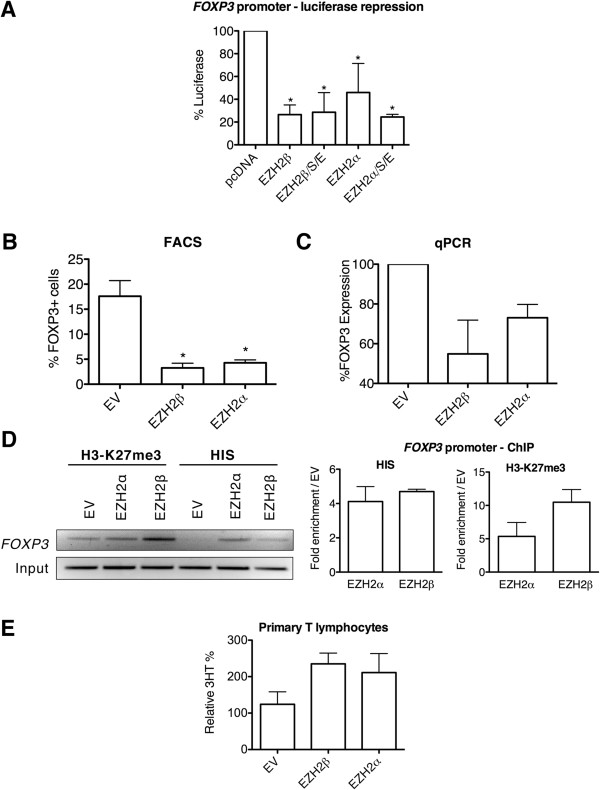
**EZH2β represses transcription through H3-K27 trimethylation of gene promoters and increases cellular proliferation.** (**A**) Luciferase values are shown relative to control (pcDNA) upon nucleofection of EZH2β or with co-factors SUZ12 and EED (EZH2β/S/E) in cells with an integrated FOXP3 luciferase reporter. EZH2α alone or with co-factors SUZ12 and EED (EZH2α/S/E) is included. *P <0.05. (**B**) Quantification of flow cytometry analysis of primary mouse naïve T cells transduced with empty vector, EZH2β or EZH2α for *FOXP3* expression. *P <0.05. (**C**) qPCR of *FOXP3* expression in primary mouse naïve T cells indicates that transcription is reduced by transduction of EZH2β and EZH2α. Glyceraldehyde-3-phosphate dehydrogenase and hypoxanthine phosphoribosyltransferase were used as expression controls. (**D**) ChIP assay of H3-K27me3 on the *FOXP3* promoter in primary mouse naïve T cells. Transduction with EZH2α or EZH2β increases H3-K27me3 on the *FOXP3* promoter relative to empty vector control. ChIP performed using an antibody against the HIS-tag demonstrates that only the EZH2α- and EZH2β-infected cells amplify a band to indicate their presence on the *FOXP3* promoter, whereas empty vector-infected cells serve as a negative control. A representative gel is shown from triplicate experiments with associated qPCR quantification. These results reveal that novel EZH2 isoforms can regulate gene expression through H3-K27 trimethylation of gene promoters. (**E**) Incorporation of ^3^H-thymidine in primary mouse naïve T cells transduced with empty vector, EZH2α or EZH2β after 5 days of stimulation. Representative data shown from experiments performed in duplicate, representing the mean and SD of technical triplicates. These results indicate that EZH2β increases cellular proliferation in a similar fashion as EZH2α. 3HT: ^3^H-thymidine; ChIP: chromatin immunoprecipitation; EV: empty vector; EZH2: enhancer of zeste homologue 2; FACS: fluorescence-activated cell sorting; H3-K27me3: trimethylation of histone 3 at lysine 27; HIS: histidine; qPCR: quantitative polymerase chain reaction.

Our experiments demonstrated that EZH2β alone or when combined with obligate PRC2 complex partners (SUZ12 and EED) significantly represses luciferase activity in JFOXP3-E1 FLP compared to transfection with empty vector alone. Compared with empty vector, EZH2 β had luciferase expression of 26.53 ±8.53% when alone and 28.60 ±17.23% when in complex, which is equivalent to a 73.7% reduction in promoter activity when alone. EZH2α was included in these experiments as a comparison and also repressed luciferase expression. EZH2α had luciferase expression of 45.97 ±25.45% and 24.46 ±2.36% compared with empty vector, alone and in complex, respectively (Figure 4A), corresponding to a 54.03% reduction in promoter activity when alone. Thus, using this engineered cell reporter system, we conclude that, *in vitro*, EZH2β displays key functional properties that are expected from a histone code writer. Equally important, these results demonstrate that various EZH2 proteins can achieve the gene silencing function previously attributed to a single HMT protein.

In light of these results, we subsequently sought to gain insight as to whether this process is also operational *in vivo* in primary cells by evaluating the role of EZH2β in the regulation of *FOXP3* expression in isolated murine T lymphocytes. As these primary cells are notoriously difficult to transfect or infect with most of the viruses used for *ex vivo* gene transfer, we isolated lymphocytes from a mouse line transgenically expressing the adenoviral receptor (CAR transgenic mouse, Taconic, model 4285) that are amenable to adenoviral-mediated transduction. Thus, naïve CD4+ splenocytes were isolated from the CAR transgenic mouse and infected with EZH2β, EZH2α or control empty adenoviruses. Primary naïve murine CD4+ lymphocytes transduced with EZH2β did not express *FOXP3* upon stimulation when compared with cells transduced with empty vector (Figure 4B and Additional file 2: Figure S2), indicating that recruitment of EZH2 to the *FOXP3* core promoter results in specific and persistent silencing of *FOXP3* expression. This result was also observed for EZH2α. Compared with 17.6 ±3.12% of *FOXP3*-expressing cells under control conditions, EZH2β overexpression reduced the number of *FOXP3*-expressing cells to 3.26 ±0.94%, and EZH2α reduced this population to 4.28 ±0.58%. Complementary qPCR assay detected a reduction of *FOXP3* transcription of 45.1 ±16.7% by EZH2β and 26.9 ±6.9% by EZH2α compared with empty vector (Figure 4C). Furthermore, in these experiments, EZH2β overexpression led to increased levels of EZH2β and H3-K27me3 bound to the *FOXP3* core promoter, which was also found with EZH2α overexpression (Figure 4D).

Through the use of two well-defined systems specially engineered to analyze EZH2-mediated gene silencing in lymphocytes (Jurkat-FLP and primary CD4+ splenocytes), we demonstrate that EZH2β is capable of gene repression that is mediated by trimethylation of H3-K27, indicating that EZH2β behaves as a histone code writer in a manner which is highly similar to the conventional EZH2α protein. These results suggest that both EZH2 proteins share mechanisms and potentially regulate similar cellular processes and gene targets. Thus, we tested these ideas by first performing functional cell assays, and subsequently, through the generation of genome-wide expression profiles for these EZH2 proteins.

### Expression of EZH2β stimulates cellular proliferation

EZH2 is among the best-characterized epigenetic regulators which, when overexpressed, increases proliferation and functions as an oncogene. Consequently, we investigated whether the new EZH2β isoform is functional in cell biological assays using cell proliferation as a model. We performed these experiments in naïve primary lymphocytes transduced with empty vector, EZH2β, or EZH2α. Figure 4e shows that overexpression of EZH2β and EZH2α results in an increase in cellular proliferation compared with the control empty vector. This functional analysis is congruent with the localization of these proteins to actively proliferating cell populations (Figure 2D) and with our data from genome-wide expression analyses, shown below, which support that both EZH2 isoforms regulate pro-proliferative gene targets. Taken together, these data indicate that EZH2β is functional in well-established cell biological assays.

### Expression of EZH2β gives rise to a unique genome-wide transcriptional profile

**Figure 5:**
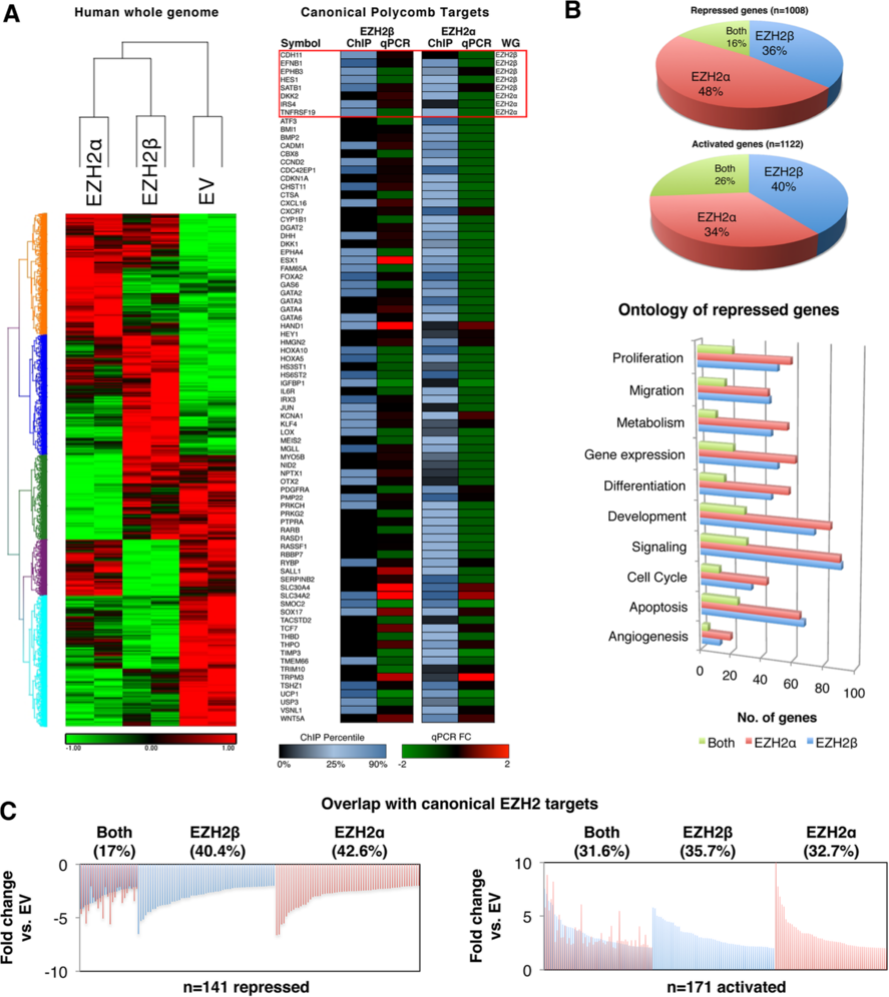
**PRC2/EZH2β governs a unique repressive program compared to conventional PRC2/EZH2α.** (**A**) Left: Genome-wide expression profiling was performed using epithelial cells transduced with EZH2α and EZH2β using Affymetrix Human Gene 1.0 ST Array. Significantly altered probes (*P* <0.05) were selected and visualized by cluster analysis. Right: A subset of known and well-characterized Polycomb targets were assessed by chromatin immunoprecipitation array using an antibody against HIS-epitope-tagged EZH2 isoforms in epithelial cells transduced with either the empty vector, HIS/EZH2β or HIS/EZH2α. Levels of binding were normalized to input controls for each of the three conditions and fold changes computed against empty vector control. Fold changes are presented according to percentile rank from 0 (unbound) to light blue (>90% percentile) of the isoform dataset. ChIP experiments were performed in duplicate with a representative dataset shown above. Targets identified as EZHβ- or EZH2α-specific from the whole genome experiment in Figure 5A-left are boxed and labeled. Comparison with expression data generated by qPCR from the same samples reveals that the majority of the target bounds by each isoform are repressed. (**B**) Comparative quantification of the percentage of uniquely repressed and activated gene targets regulated by each isoform individually or both isoforms is indicated (*P* <0.05, log_2_ fold change >−2). The ontological classification of targets uniquely repressed by each isoform individually or in conjunction with the other isoform is also shown. (**C**) Comparative quantification of the percentage of uniquely repressed and activated gene targets against a subset of canonical EZH2 targets as determined by an independent ChIP-seq dataset that used an antibody predicted to cross-react with multiple EZH2 isoforms. ChIP: chromatin immunoprecipitation; EV: empty vector; EZH2: enhancer of zeste homologue 2; qPCR: quantitative polymerase chain reaction.

Expression profile experiments offer a genome-wide level reporter assay to investigate whether EZH2α and EZH2β possess common or divergent functions. This experiment was chosen because EZH2β expression follows, in most cases, the expression pattern of EZH2α in the majority of tissue types studied. EZH2 is a known oncogene for a large number of tissues, including pancreatic cancer. Thus, we used a pancreatic epithelial cell system combined with adenoviral-mediated introduction to overexpress each isoform in an attempt to model the effects of pathological overexpression of each EZH2 isoform on gene repression. For this purpose, we performed Affymetrix GeneChip Human Gene 1.0 ST arrays, which showed that of the 28,869 well-annotated genes assessed, 366 unique targets (36.3% of total repressed) were uniquely repressed by EZH2β (*P* <0.05 and log_2_ fold change >−2 for EZH2β, *P* >0.05 for EZH2α, Figure 5). EZH2α-generated expression profiles displayed a downregulation of 480 targets (47.6% of total repressed, *P* <0.05 and log_2_ fold change >−2 for EZH2α, *P* >0.05 for EZH2β, Figure 5). Notably, 162 targets (16.1% of total repressed) were repressed by both EZH2α and EZH2β (Figure 5, *P* <0.05, log_2_ fold change >−2 for both). Both isoforms also induced upregulation in the expression of a significant number of targets, which may reflect indirect effects mediated by the repression of upstream regulators. Of the genes assayed, 444 (39.6% of total activated) were activated by EZH2β, 382 (34%) by EZH2α and 296 (26.4%) by both isoforms. Therefore, as demonstrated by the overall array data, the novel EZH2β isoform described here is responsible for the expression pattern of its own unique set of genes, in addition to a group of common targets with EZH2α.

Repressed genes were organized into ontological categories using Ingenuity Pathways Analysis (IPA)-based classifications (Figure 5B). Notably, EZH2β was found to regulate genes involved in key cellular functions including proliferation, differentiation and angiogenesis, which were previously attributed only to EZH2 isoform. This concept was better visualized by cross-validating our expression data with a subset of canonical EZH2-regulated targets as identified by a previously reported EZH2 chromatin immunoprecipitation-sequencing (ChIP-seq) dataset in a different cell line [35], generated using an antibody that, according to our data, recognizes both the EZH2α and EZH2β isoforms. Cross-reference of significantly repressed and activated genes parsed from our genome-wide expression data (Figure 5C) with this independent subset of targets demonstrates a division pattern similar to that observed in the transcriptional profiles. As such, from a large subset of genes previously thought to be regulated by a single EZH2 HMT, we determined the overlap between the isoform-specific targets we identified and this subset of canonical EZH2 targets. Figure 5A-right depicts both occupancy and expression measured on a subset of well-validated canonical Polycomb targets that have previously been shown to be regulated by EZH2α. The box highlights a subset of targets that were identified as EZH2β or EZH2α-specific from our whole genome assay (Figure 5A-left). Thus, although each isoform possesses similarity in terms of ontological functions, mediation of these functions appears to be executed through the repression of primarily unique, although sometimes common, targets.

**Figure 6:**
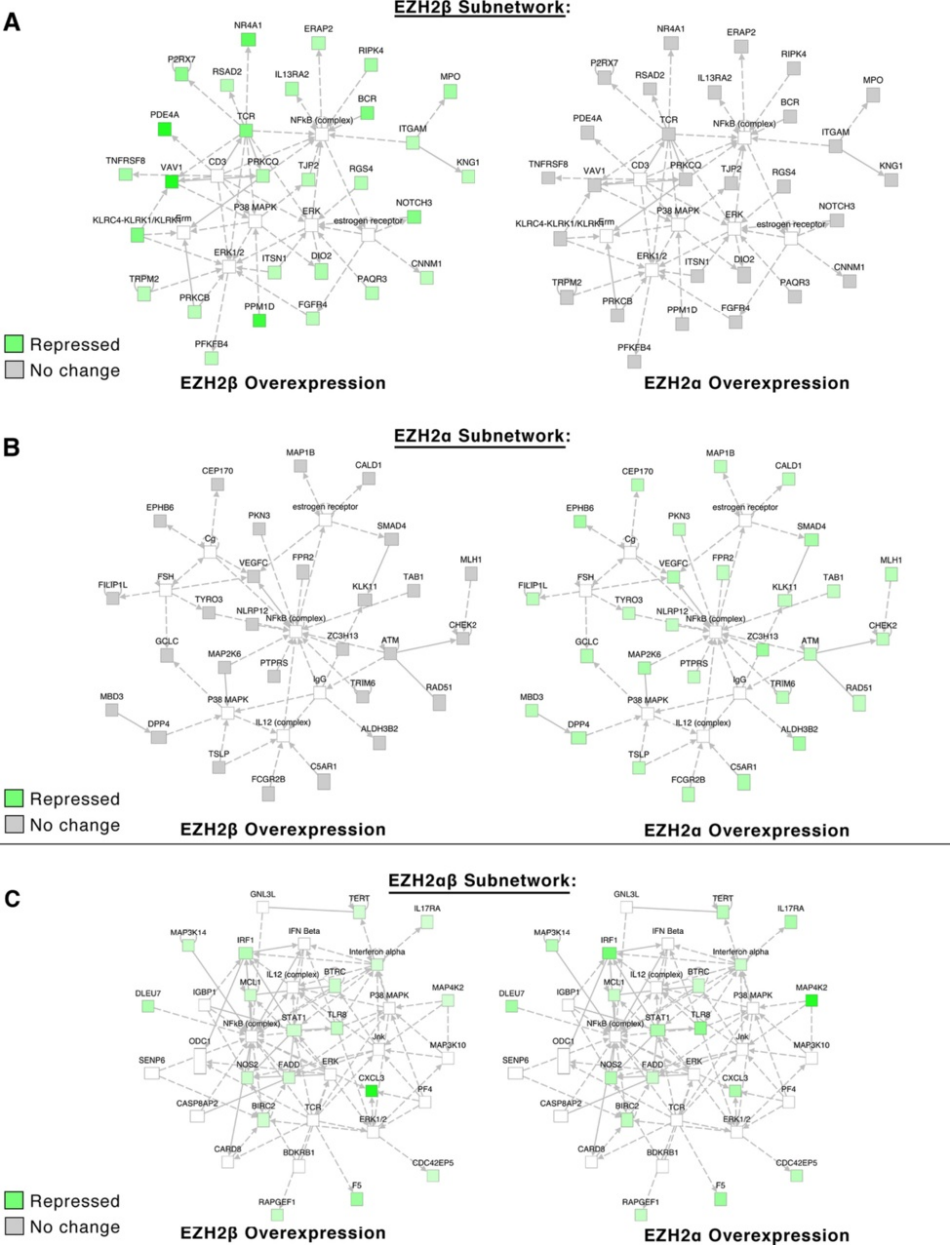
**EZH2 isoforms can regulate gene expression genome-wide through defined subnetworks.** To test if EZH2β- or EZH2α-specific genes form interdependent, unique subnetworks, Affymetrix data generated in A was parsed for targets uniquely and significantly repressed (P <0.05, log2 fold change >−2) by EZH2β, EZH2α or both compared with empty vector. No change is defined as P >0.05 and log2 fold change between 1.5 and −1.5. Subnetworks were reconstructed using an IPA-propriety algorithm. While a multitude of subnetworks were generated, a high scoring representative example network for each condition is shown. (**A**) IPA-assisted subnetwork analysis indicates that EZH2β participates in the regulation of genes involved in several ontological categories, including cellular function and maintenance as well as hematological system function. Overexpression of EZH2β results in significant repression whereas EZH2α overexpression fails to produce the same repressive effects in this particular subnetwork. (**B**) Similar subnetwork analysis of expression data for enrichments of biological function mediated by EZH2α indicates that this protein regulates targets associated with proliferative responses and cell cycle regulation. (**C**) Subnetwork analysis of expression data for enrichments of biological function mediated by both EZH2β and EZH2α indicates enrichment of targets involved in cell death and survival and cell signaling. Combined, these data demonstrate that that novel EZH2 isoforms can regulate gene expression genome-wide through unique and shared targets that are interconnected to form defined subnetworks. Note that, although both EZH2 isoforms can often regulate different genes represented by the examples (A, B and C), the subnetworks formed by these genes are ontologically known to participate in similar processes (B). This knowledge not only is congruent with the ability of both EZH2 proteins to regulate cell growth as revealed by our cell biology assays (Figure 4E), but also expands the potential functional association of these isoforms. EZH2: enhancer of zeste homologue 2.

IPA-based network analysis identified a number of subnetworks of interdependent genes enriched for particular functions and/or participation in disease processes. EZH2β, for instance, was able to uniquely repress a subnetwork enriched for functions in cellular maintenance and function as well as hematological system development and function (Figure 6A). Overexpression of EZH2α, however, led to no significant alteration of these targets. EZH2α overexpression resulted in the significant repression of a subnetwork of targets that associates to the regulation of cellular growth, cell cycle and proliferation (Figure 6B). Again, EZH2α repressed many of these targets uniquely without apparent contribution from EZH2β. However, subnetwork enrichment for function in cell death survival displayed equal repression by either isoform (Figure 6C). Thus, these data indicate that although biochemically quite similar at the level of nuclear localization, transcription and interaction with critical co-factors, each isoform displays a preferential gene expression pattern, which, according to our ontological analyses, supports their participation in a large number of shared biological functions.

## Discussion

The human *EZH2* gene was originally isolated in a screen for proteins which interact with Vav1, a human proto-oncogene [36,37]. Notably, although most Polycomb functions have been attributed to require the enzymatic activity of PRC2, recent data indicate that other related enzymes may possess redundant or overlapping functions with EZH2, such as EZH1 [10,38]. Despite these advances, most Polycomb experiments are designed with the paradigm that EZH2α is the sole H3-K27me3 methyltransferase. Thus, it has become essential to explore the isoform complexity of the EZH2 family of proteins. Consequently, the goal of the current study has been to address this important gap in the existing knowledge.

*Drosophila* possess only one E(z) gene, whereas vertebrates possess two paralogs: EZH1 and EZH2, with gene duplication occurring early in evolution, as two paralogs have been identified in zebrafish [10]. Evidence for alternative splicing is evident even in the ancestral E(z) gene with observed expansions and reductions in the progression from invertebrate to vertebrate. Although gene duplication of HMT genes, as observed with Ez proteins and other HMTs, such as Suv4-20 h1/h2 and Suv39h1/h2 [39], appear to serve redundant functions, the early expansion of EZH2 through alternative splicing hints at a neofunctionalization phenomenon. The preservation of alternative splicing events from invertebrates to vertebrates supports an evolutionary model in which pressures were high to maintain a diverse pool of EZH2 proteins to facilitate precise regulation of repressive programs.

We have characterized the alternative splicing and translation of the *EZH2* locus to yield a minimum of two distinct functional HMTs: EZH2α, a known enzyme, and EZH2β, a new enzyme. Biochemical characterization of EZH2β indicates that it exhibits a similar tissue expression pattern as EZH2α and that this isoform is widely expressed in human tissues with particularly high levels of expression in tissues dependent on replenishment from a progenitor pool of multipotent cells, such as the thymus and testes. Multiple EZH2-positive bands have been observed by Southern and western blot in previous studies [25,40,41], but were often labeled as artifact. However, our investigation is the first to positively confirm and characterize two distinct isoforms using antibodies designed to distinguish between each protein. Furthermore, we demonstrate that EZH2β is localized exclusively to the nucleus and capable of partnering with obligate Polycomb co-factors SUZ12 and EED to form the minimal PRC2 complex necessary to permit enzymatic activity of the protein. Since the identification of its mammalian homologue, a number of EZH2 transcripts have been identified by genomic sequencing efforts, supporting the existence of a family of EZH2 proteins that mediate mammalian gene repression. Extensive future characterization will be required to determine the precise role of each protein variant in gene repression.

PRC2-EZH1 and PRC2-EZH2α regulate a largely overlapping set of genes, albeit through different mechanisms; PRC2-EZH1 possesses greatly reduced HMT activity compared with PRC2-EZH2α [10]. Both EZH2α and EZH2β are capable of repressing *FOXP3* expression *in vitro*, in a manner that is increased by transfection with obligate co-factors SUZ12 and EED. More importantly, using primary mouse naïve T cells, we demonstrate that both isoforms are able to occupy the *FOXP3* promoter with resultant increases in H3-K27me3 and repression of *FOXP3* expression, suggesting identical mechanisms of repression. These results highlight the importance for future studies to consider the relative contributions of both isoforms in the regulation of gene repression.

We demonstrate that EZH2β represses a predominantly unique subset of gene targets from EZH2α with a much smaller percentage of redundant targets than observed between EZH paralogs EZH1 and EZH2 [10]. Although ontology reveals that both isoforms participate in a similar repertoire of biological processes, subnetwork analysis of significantly repressed genes indicates that each isoform regulates distinctive gene networks within process categories. Furthermore, comparison of EZH2α and EZH2β targets with published ChIP-seq data performed with an antibody that fails to discriminate between isoforms reveals a similar pattern as gene expression data, with each isoform possessing a larger subset of unique rather than redundant targets [35]. The primary difference between the two isoforms is the 39 amino acid insert absent in EZH2β compared with EZH2α. Examination of this insert reveals the presence of potential sites of post-translational modification, including an (ST)-Q motif, which have the potential to be targeted by kinases that participate in a variety of cellular processes including DNA replication and repair. Thus, these data serve as the foundation for future studies aimed at investigating how post-translational modifications can contribute to impart functional specificity of each isoform. Coupled with biochemical data, these studies indicate that EZH2α and EZH2β are capable of forming distinct repressive complexes that mediate the repression of unique gene networks within a wide variety of biological processes already characterized for PRC2, including proliferation, migration and differentiation, among others [42].

Whole genome gene expression data reveals enrichment for cell cycle and proliferation targets. Overexpression of either isoform in naïve T cells results in increased cellular proliferation. Additionally, immunohistochemistry of total EZH2 versus EZH2β reveals that EZH2β is localized primarily to developing spermatogonia whereas total EZH2 expression is localized throughout the spermatogonia and spermatocytes. As spermatogonia undergo mitosis, compared to the meiosis occurring in spermatocytes, a potential role for EZH2β in the regulation of cell cycle transitions is likely [43]. Thus, our studies offer a solid rational and build the trajectory for future careful studies aimed at deciphering the role of EZH2 isoforms at the G1/S and G2/M transition points, as well as the type of post-translational modifications, that can regulate these processes.

## Conclusions

Thus far, the functions of EZH2 have been ascribed entirely to isoform EZH2α. The current body of literature will require revision to address the relative contribution of EZH2 isoforms to the biochemical, cellular and pathobiological functions under study. Furthermore, the contribution of alternative splicing to the regulation of HTMs and their function furthers our understanding of the complexity of regulatory mechanisms underlying the operation of the histone code. As a result of these findings, a new paradigm of Polycomb-mediated repression must be considered in which cells are armed with a multitude of repressive complexes to regulate distinct gene networks, exponentially increasing the plasticity of the system to meet the broad spectrum of functions required in development, growth and maintenance of biological systems.

## Methods

### Plasmids and recombinant adenovirus

The search for EZH2-related proteins was performed by comparing the human EZH2 SET domain protein sequence (GenBank: BC010858) against the Expressed Sequence Tag database using the BLAST and the UniGene programs from the National Center for Biotechnology Information (National Institutes of Health, Bethesda, MD, USA). This comparison indicated the presence of the EZH2β-encoding sequence (NCBI: NM_152998.2). The exact sequences matching this entry as well as other PRC2 proteins, such as SUZ12 (GenBank: BC015704) and EED (GenBank: BC068995), were verified by sequencing and analysis of publically deposited cDNAs. Standard molecular biology techniques were used to clone full-length EZH2α, EZH2β, SUZ12 and EED into pcDNA3.1/HIS (Invitrogen, Carlsbad, CA, USA). All constructs were verified by sequencing at the Mayo Clinic Molecular Biology Core Facility. QuickChange Site-Directed Mutagenesis was performed as suggested by the manufacturer (Agilent Technologies, Santa Clara, CA, USA). Silent mutations were made to delete endogenous HindIII and XbaI restriction enzyme sites to permit passage of EZH2α and EZH2β cDNAs into pacAd5 CMV K-N pa shuttle vector. Epitope-tagged (6XHis-Xpress) EZH2α and EZH2β were generated as recombinant adenoviruses by the Gene Transfer Vector Core at the University of Iowa. Empty vector (pacAD5 CMV) was used as the experimental control.

### Human tissue RNA panel

Human total RNA for 22 major organs and tissues was commercially obtained from Ambion (Austin, TX, USA) and Stratagene (Agilent). cDNA was generated from 1 μg RNA using SuperScript^T^ III enzyme (Invitrogen) according to manufacturer’s instructions. cDNA concentrations were assessed via internal housekeeping gene glyceraldehyde-3-phosphate dehydrogenase or hypoxanthine phosphoribosyltransferase. PCRs were performed with the following cycle conditions: 30 to 35 cycles of 94°C for 15 s, 50°C for 30 s, and 72°C for 2 min using 1 to 2 μl of cDNA product. Amplified products were electrophoresed on 1.5% agarose gels, digitally imaged, and quantified with ImageJ (National Institutes of Health, Bethesda, MD, USA). Primers were synthesized by Integrated DNA Technologies (Coraville, IA, USA). PCR primers may be found in Additional file 3: Table S1.

### Western blot analysis

Samples were run on 4% to 20% (Lonza, Walkersville, MD, USA), 6% or 10% SDS-PAGE gels and electroblotted onto polyvinylidene difluoride membranes (Millipore, Billerica, MA, USA). The membranes were blocked in 5% bovine serum albumin or milk in Tris buffered saline with Tween (TBST) for 1 h at room temperature. The blots were incubated overnight at 4°C with primary antibody. After repeated washes in TBST, horse radish peroxidase -conjugated anti-rabbit or mouse IgG secondary antibody (1:2,000 to 5,000) was added for 1 h at room temperature. Blots were developed by Pierce ECL Chemiluminescent Substrate (Thermo Scientific, Rockford, IL, USA). Human tissue lysates were procured from Calbiochem (Millipore) as a ready-to-probe INSTA-blot. Approximately 20 μg of lysate was loaded per tissue with loading controlled via amido black straining by the manufacturer. The blot was incubated overnight with EZH2β (purified, 1:2,000) and subsequently stripped and re-incubated with β-actin (1:1,000; Sigma, St. Louis, MO, USA).

### Synthesis, purification and validation of EZH2α and EZH2β antibodies

A 21-mer peptide bridging across the large insert region missing from EZH2β compared to EZH2α was synthesized, high performance liquid chromatography-purified and conjugated to keyhole limpet hemocyanin by the Mayo Clinic Protein Core. For the EZH2α antibody, a 21-mer peptide was synthesized that localized to the insert region. Subsequently, a rabbit was immunized with the peptide, and test and final bleeds were performed by Cocalico Biologicals (Reamstown, PA, USA). For the antibody that recognizes both EZH2α and EZH2β, a 21-met peptide in a region conserved between the two proteins was synthesized. The anti-serum was affinity purified using the Protein A IgG Purification Kit according to the manufacturer’s protocol (Pierce Biotechnology, Rockford, IL, USA). To test the specificity of the antibodies, Chinese hamster ovary epithelial cells were transfected with a histidine-tagged (HIS)/EZH2α and HIS/EZH2β. Whole cell lysates (30 μl) and pancreatic cell lines (30 μg) were resolved on 4% to 20% SDS-PAGE gels, and probed with whole sera of EZH2α (1:200), EZH2β (1:200) and EZH2αβ (1:200). Blots were stripped and re-probed with Omni-probe (D-8) (1:1,000; Santa Cruz Biotechnology, Santa Cruz, CA, USA) to ensure equal loading.

### Immunoprecipitation

Panc1 epithelial cells were plated at a cell density of 1 × 10^6^ cells/100 mm dish and transduced with epitope-tagged (6XHis-Xpress) EZH2α, EZH2β or empty vector at multiplicity of infection (MOI) 150. Subconfluent cells were lysed in a buffer containing 20 mM Tris-Cl at pH 8.0, 100 mM NaCl, 1 mM EDTA, 0.5% Nonidet P-40 and a protease inhibitor tablet (Roche, San Francisco, CA, USA). Proteins were immunoprecipitated as previously described using 10 μg of Omni-probe (D-8) (Santa Cruz Biotechnology) [44]. Resulting complexes were resolved on a 6% or 10% SDS-PAGE gels, using antibodies against SUZ12 (1:1,000; Cell Signaling, Beverly, MA, USA) and EED (1:1,000; Cell Signaling). Membranes were stripped and incubated with Omni-probe (D-8) (1:1,000; Santa Cruz), to ensure equal loading of precipitated EZH2 proteins. A 5% input control of whole cell lysates under all conditions was included to ensure the presence of uniform levels of the proteins of interest.

### Cell culture, immunofluorescence and confocal microscopy

Cell lines were obtained from the American Type Culture Collection (ATCC, Rockville, MD, USA) and maintained according to their recommendations. Immunofluorescence and confocal microscopy were performed as previously described [44]. Panc1 cells were plated in eight-chamber glass slides at a density of 5 × 10^4^ cells/chamber and transduced with epitope-tagged (6XHis-Xpress) EZH2α, EZH2β or empty vector at MOI 150. Primary antibodies were used at the following dilutions: EZH2α (1:50; described above), EZH2β (1:50; described above), EZH2 (1:200; Cell Signaling) and Omni-probe (D-8) (1:250; Santa Cruz). Images were obtained at 100× magnification. Frozen cryosections of human testis (5 μm) were purchased from Zyagen (San Diego, CA, USA). Sections were fixed in ice-cold acetone for 10 min and rehydrated in PBS for 3 min. Endogenous peroxidase activity was quenched using a 3% hydrogen peroxide in methanol for 20 min (Sigma). Avidin/Biotin blocking was performed using a kit from Vector Laboratories (Burlingame, CA, USA). Tissues were blocked in CAS Block for 1 h (Invitrogen) prior to overnight incubation at 4°C in primary antibody. Dilutions were as follows: EZH2β (1:200; described above) and EZH2 (1:200; Cell Signaling). Sections were subsequently washed in PBS and incubated in biotinylated goat anti-rabbit secondary antibody (Vector Laboratories) for 30 min. Samples were incubated in Alexa Fluor-488-streptaviding conjugate (Invitrogen). Sections were counterstained with Hoescht. Images were obtained at 10× magnification.

### Microarray, validation and subnetwork constructions

BxPC3 epithelial cells were plated at a density of 1 × 10^6^ cells/100 mm dish and transduced with empty vector, EZH2α or EZH2β (Ad5CMV) at an MOI of 150. RNA was prepared as previously described 48 h after transduction [44]. Experiments were performed from pooled biological triplicates in technical duplicates. The transduction efficiency of these cells at MOI 150 is 81.3 ±1.99% as determined by transduction with GFP adenovirus. Global gene expression profiling was carried out at the Microarrays Facility of the Research Center of Laval University CRCHUL using the Affymetrix Human Gene 1.0 ST arrays (28,869 well-annotated genes and 764,885 distinct probes). Intensity files were generated by Affymetrix GCS 3000 7 G and the GeneChip Operating Software (Affymetrix, Santa Clara, CA, USA). Data analysis, background subtraction and intensity normalization was performed using robust multiarray analysis [45]. Genes that were differentially expressed along with false discovery rate were estimated from t test (>0.005) and corrected using Bayes approach [46,47]. Data analysis, hierarchical clustering and ontology were performed with the OneChanelGUI to extend affylmGUI graphical interface capabilities [48] and Partek Genomics Suite, version 6.5 (Partek Inc., St. Louis, MO, USA) with analysis of variance analysis. A cutoff of expression log_2_ fold change of two and *P* <0.05 was set to identify molecules whose expression was significantly differentially regulated. EZH2β and EZH2α baseline transcript levels were assessed compared to overexpression by qPCR to assure that each isoform was expressed at approximately equivalent levels (Additional file 4: Figure S3A). Additionally, a small subset of targets was validated by qPCR (Additional file 4: Figure S3C).

Selected probes and their fold changes were loaded into IPA Software (Ingenuity Systems. Each identifier was mapped to its corresponding object in the Ingenuity Knowledge Base. These molecules, called Network Eligible molecules, were overlaid onto a global molecular network developed from information contained in the Ingenuity Knowledge Base. For the purposes of network reconstruction, a log_2_ fold change of two was used. Networks of Network Eligible molecules were then algorithmically generated based on their connectivity. The functional analysis of a network identified the biological functions and/or diseases that were most significant to the molecules in the network. The network molecules associated with biological functions and/or diseases in the Ingenuity Knowledge Base were considered for the analysis. Right-tailed Fisher’s exact test was used to calculate a *P*-value determining the probability that each biological function and/or disease assigned to that network was due to chance alone.

### Flp-in system, transfection and luciferase assays

The human *FOXP3* core promoter containing −511 bp from transcription start site was amplified by PCR using *FOXP3* promoter sequence-specific primers from position −511 to +176. The genomic DNA extracted from CD4^+^ T cells of a healthy donor was used as a template. The PCR product was subcloned in the pGL3 basic vector (Promega, Madison, WI, USA). Similarly, the *FOXP3* core promoter plus the first enhancer (E1) containing −511 bp to +2,738 was also amplified by PCR and subcloned in the pGL3 basic vector (Promega). The Flp-In system (Invitrogen) was used for the generation of a stable human *FOXP3* core promoter and FOXP core +E1 promoter Flp-In-Jurkat. Flp-In-Jurkat cells (Invitrogen) were co-transfected with *FOXP3* core or *FOXP3* core + E1 in a pcDNA5/ FLP recombination target (FRT) vector and a FLP-recombinase vector (pOG44) (pOG44:*FOXP3* core or *FOXP3* core + E1/pcDNA5/FRT ratio = 9:1), resulting in a stable integration of the gene of interest at the FRT-site in the genome. For the selective growth test, individual cells were grown in 24-well plates. The culture medium was supplemented with hygromycin at 250 μg /ml or 100 μg/ml. Two million *FOXP3* core and *FOXP3* core + E1 Flp Jurkat cells were transfected using the Amaxa Cell Line Nucleofector Kit V for Jurkat cells according to the optimized protocol provided with the kit. Two micrograms of plasmid DNA for EZH2α, EZH2β, SUZ12 and EED were used in the nucleofection procedure. Luciferase assays were done following the manufacturer’s recommendations (Promega). Data represent the mean and SD of three independent experiments (*P <0.05).

### Adenoviral transduction and flow cytometry

The CAR transgenic mouse was obtained through the NIAID Exchange Program, NIH: Balb/cJ[Tg]CARdelta1-[Tg]DO11.10 mouse line #4285 [49,50]. Murine naïve CD4+ splenocytes were isolated using a combination of magnetic separation kits (Miltenyi Biotec, Auburn, CA, USA). Sequential use of the CD4+CD25+ regulatory T cell isolation kit and the CD4+CD62L+ T cell isolation kit resulted in naïve *FOXP3*-negative T cells used for *in vitro* induction of *FOXP3*. Naïve T cells were isolated from the CAR transgenic Balb/cJ[Tg]CARdelta1-[Tg]DO11. Cells were activated for 48 h with empty vector, EZH2α or EZH2β at an MOI of 250. The transduction efficiency of these cells as determined by flow cytometry with propidium iodide exclusion using GFP adenovirus is 89.4 ±2.1%. Cells were activated under the typical stimulation conditions for 3 days and processed for ChIP and qPCR to determine methylation of H3K27me3 marks at the *FOXP3* core promoter and levels of *FOXP3* expression, respectively. Flow cytometry was used to look at levels of *FOXP3* expression within the CD4+ population across four biological replicates. Intracellular staining procedures for *FOXP3* were followed using the application notes from Alexa Fluor 488 anti-mouse/rat/human *FOXP3* (BioLegend, San Diego, CA, USA). For qPCR analysis, biological triplicates were pooled and analyses performed in technical duplicate. Data represent the mean and SD of four independent experiments (*P <0.05).

### T cell stimulation

*In vitro* activation of the isolated T cells followed similar conditions among the different cell types. Anti-CD3, OKT3 (eBioscience, San Diego, CA, USA) for the Jurkat cells, 145-2C11 (BD Biosciences, San Jose, CA, USA) for the mouse T cells, and UCHT1 (BD Biosciences) for the human T cells was platebound at 2 μg/ml. Soluble anti-CD28 (BD Biosciences) at 2 μg/ml plus 100 units/ml IL-2 was added to the cultures throughout the incubation period. Human transforming growth factor beta-1 recombinant (AUSTRAL, San Romano, CA, USA) at a concentration of 5 ng/ml was used to generate adaptive Treg cells.

### Chromatin immunoprecipitation assays

ChIP assays were performed as previously described using H3-27me3 (Cell Signaling) and Omni-probe (D-8) (Santa Cruz) antibodies [51]. Primers used to analyze the *FOXP3* promoter are listed in Additional file 3: Table S1. For the Polycomb target screen, mRNA and ChIP samples were processed from BxPC3 epithelial cells as described above and used with the Human Polycomb and Trithorax Target Genes ChIP PCR Array (SA Biosciences, Valencia, CA, USA). ChIP were performed in biological duplicate and of the 84 targets present on the array, 78.6% (66 out of 84) were occupied by EZH2α, serving as an internal positive experimental control. Expression profiling was performed in biological triplicate with the averaged values reported.

### ^3^H-thymidine incorporation proliferation assay

Naïve T cells from a CAR D011.10 mouse were isolated and transduced with empty vector, EZH2α and EZH2β as described above. Cells were plated at 6.6 × 10^5^/ml in complete Roswell Park Memorial Institute medium containing αCD28 at 2 μg/ml plus 100 units/ml IL-2, and 200 μl was added per well to a 96-well round bottom plate coated with αCD3 at a concentration of 2 μg/ml. Five days after plating, 20 μl of ^3^H-thymidine (6.7 Ci/mmol NET-027) at a 1:20 dilution in complete Roswell Park Memorial Institute medium (1.0 μCi) was added to each well and incubated for approximately 18 h. Cells were harvested and counted on the microtiter plate counter.

### Bioinformatics and statistical analysis

Bioinformatics-assisted splice-mapping of the human *EZH2* locus was performed using AceView [52]. An evolutionary dendrogram of common invertebrate and vertebrate EZH2 isoforms was created using the Geneious Tree Builder with a BLOSUM62 matrix, free end global alignment with a gap open penalty of 12 and a gap extension penalty of 3 (no outbound group selected). Predicted EZH2 splice variant sequences were curated from National Center for Biotechnology Information. Statistical analyses were performed using Graphpad Prism (La Jolla, CA, USA). Descriptive analyses including means and SDs were performed in normally distributed data. One-way analysis of variance with Tukey’s post-hoc test was utilized to determine statistically significant observations. A *P*-value of <0.05 was considered as statistically significant.

## Abbreviations

BLAST: Basic Local Alignment Search Tool; bp: Base pair; ChIP: Chromatin immunoprecipitation; CMV: Cytomegalovirus; EED: Embryonic ectoderm development; EZH2: Enhancer of zeste homologue 2; GFP: Green fluorescent protein; H3-K27me3: Trimethylation of histone 3 at lysine 27; HIS: Histidine; HMT: Histone methyltransferase; Ig: Immunoglobulin; IL: Interleukin; IPA: Ingenuity Pathways Analysis; kDa: kiloDalton; MOI: Multiplicity of infection; PBS: Phosphate-buffered saline; PCR: Polymerase chain reaction; PRC: Polycomb repressive complex; qPCR: Quantitative polymerase chain reaction; RBBP: Retinoblastoma binding protein; RT-PCR: Reverse transcription polymerase chain reaction; SD: Standard deviation; SUZ12: Suppressor of zeste 12; TBST: Tris buffered saline with Tween.

## Competing interests

The authors declare that they have no competing interests.

## Authors’ contributions

AG, GL and RU generated the main idea of the work and developed the study design, both conceptually and methodologically. AG, PS, AM, EC and YX made substantial contributions to acquisition of data. AG, GL, PS, AM, YX, EC, JI, WF and RU contributed to analysis and interpretation of data. AG, GL, PS, AM, YX, EC, JI, WF and RU were in charge of writing the manuscript from first draft to completion. AG, GL, PS, AM, YX, EC, JI, WF and RU made comments, suggested appropriate modifications and corrections that were included in the final version of this article, which all authors read and approved.

## Supplementary Material

Additional file 1: Figure S1 Identification of multiple EZH2-positive bands in pancreatic epithelial cells. Thirty micrograms of whole cell extracts from a subset of pancreatic cells lines were examined for EZH2α and EH2β expression and probed with whole sera of EZH2α (1:200), EZH2β (1:200) and EZH2αβ (1:200). Note that while in some instances, EZH2α and EZH2β are equally spliced, in other cases, only one isoform predominates. β-actin is used here as a loading control. Red arrows indicate bands of interest. Click here for file

Additional file 2: Figure S2 FACS analysis of FOXP3+ cells under EZH2β and EZH2α overexpression. Representative figure of raw data. Primary naïve murine CD4+ lymphocytes transduced with EZH2β did not express *FOXP3* upon stimulation when compared to cells transduced with empty vector. Bracket indicates the population of FOXP3+ lymphocytes from the total population of viable naive lymphocytes. Quantification of results reported in Figure 4B represents the average of four biological replicates. Click here for file

Additional file 3: Table S1 PCR primers. Tables of primers utilized for experiments described in this manuscript. Click here for file

Additional file 4: Figure S3 Affymetrix microarray validation. **(A)** qPCR of EZH2β and EZH2α expression in transduced BxPC3 epithelial cells was used to assess the levels of EZH2β and EZH2α transcript at baseline (empty vector control) and overexpression conditions (MOI 150). Hypoxanthine phosphoribosyltransferase was used as a housekeeping control for normalization. **(B)** Western blot of whole cells extracts from the overexpression conditions described in A probed with antibodies against EZH2α, EZH2β and HIS-tag. β-actin was used as a loading control. **(C)** To validate the results of the Affymetrix GeneChip Human Gene 1.0 ST microarray, five targets were selected for validation via qPCR. Results are presented as a scaled, comparative heatmap. Click here for file
