# Supplementary figures and images for: Functional characterization of EZH2β reveals the increased complexity of EZH2 isoforms involved in the regulation of mammalian gene expression
Source: Epigenetics Chromatin. 2013 Feb 28;6:3. doi: 10.1186/1756-8935-6-3 (PMC3606351; doi:10.1186/1756-8935-6-3)

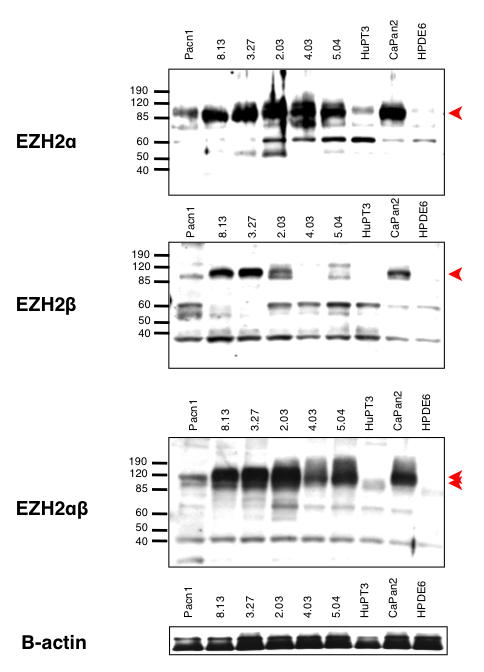

Supplement: Additional file 1: Figure S1 — Identification of multiple EZH2-positive bands in pancreatic epithelial cells. Thirty micrograms of whole cell extracts from a subset of pancreatic cells lines were examined for EZH2α and EH2β expression and probed with whole sera of EZH2α (1:200), EZH2β (1:200) and EZH2αβ (1:200). Note that while in some instances, EZH2α and EZH2β are equally spliced, in other cases, only one isoform predominates. β-actin is used here as a loading control. Red arrows indicate bands of interest. [file 1756-8935-6-3-S1.tiff]

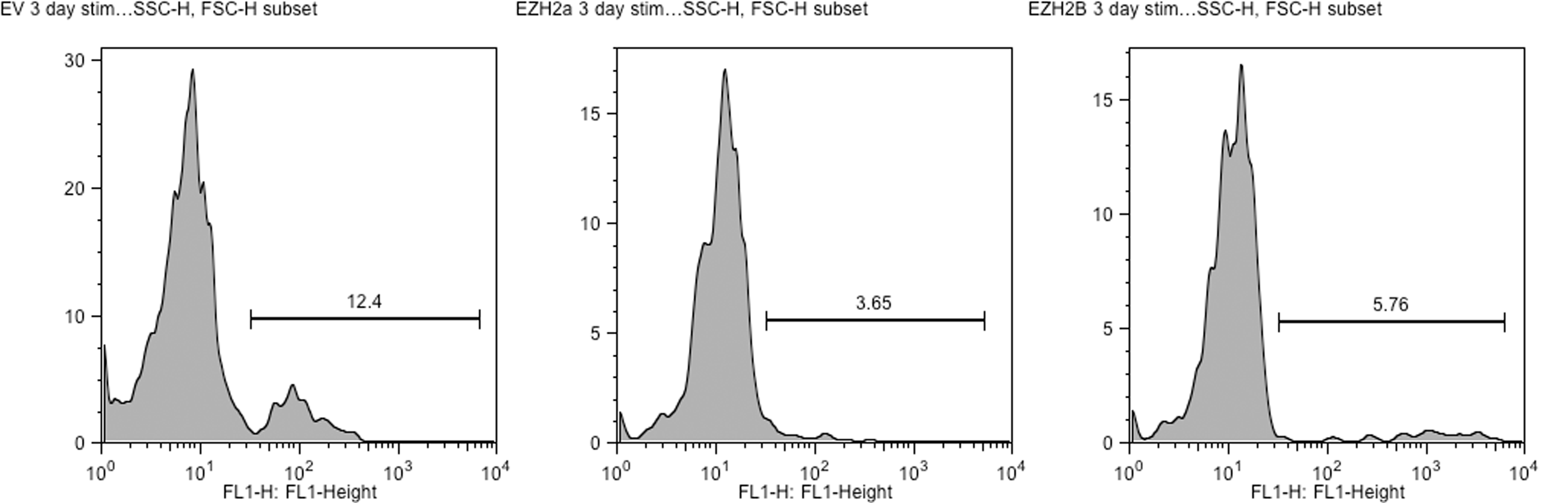

Supplement: Additional file 2: Figure S2 — FACS analysis of FOXP3+ cells under EZH2β and EZH2α overexpression. Representative figure of raw data. Primary naïve murine CD4+ lymphocytes transduced with EZH2β did not express FOXP3 upon stimulation when compared to cells transduced with empty vector. Bracket indicates the population of FOXP3+ lymphocytes from the total population of viable naive lymphocytes. Quantification of results reported in Figure 4B represents the average of four biological replicates. [file 1756-8935-6-3-S2.tiff]

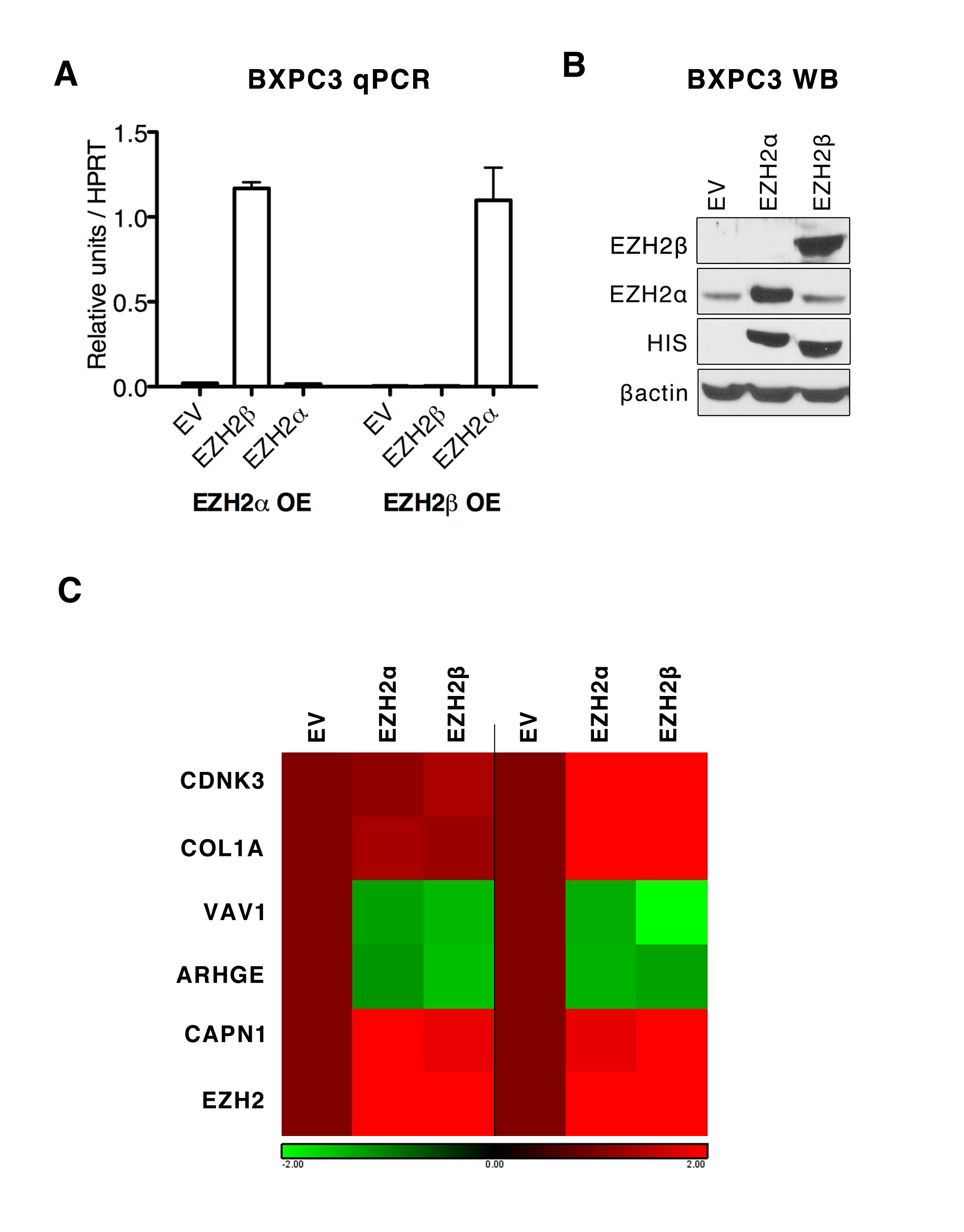

Supplement: Additional file 4: Figure S3 — Affymetrix microarray validation. (A) qPCR of EZH2β and EZH2α expression in transduced BxPC3 epithelial cells was used to assess the levels of EZH2β and EZH2α transcript at baseline (empty vector control) and overexpression conditions (MOI 150). Hypoxanthine phosphoribosyltransferase was used as a housekeeping control for normalization. (B) Western blot of whole cells extracts from the overexpression conditions described in A probed with antibodies against EZH2α, EZH2β and HIS-tag. β-actin was used as a loading control. (C) To validate the results of the Affymetrix GeneChip Human Gene 1.0 ST microarray, five targets were selected for validation via qPCR. Results are presented as a scaled, comparative heatmap. [file 1756-8935-6-3-S4.tiff]
