# Supplementary material for: Functional characterization of EZH2β reveals the increased complexity of EZH2 isoforms involved in the regulation of mammalian gene expression
Source: Epigenetics Chromatin. 2013 Feb 28;6:3. doi: 10.1186/1756-8935-6-3 (PMC3606351; doi:10.1186/1756-8935-6-3)
Supplement: Additional file 3: Table S1 — PCR primers. Tables of primers utilized for experiments described in this manuscript. [file 1756-8935-6-3-S3.docx]

**Supplemental Table I: PCR Primers**

| **TARGET** | **SPECIES** | **FORWARD (5’-3’)** | **REVERSE (5’-3’)** |
| --- | --- | --- | --- |
| EZH2 (all) | *H. sapiens* | CGAGCTCCTCTGAAGCAAAT | AACCTAGCAATGGCACAGAAA |
| EZH2α | *H. sapiens* | GCGGGACTAGGGAGTGTTC | AAAACAGTTTCATCTTCCACCA |
| EZH2β | *H. sapiens* | GGGACTAGGGAGGTGGAAGA | GCATTCACCAACTCCACAAA |
| SUZ12 | *H. sapiens* | TCGGAATCTCATAGCACCAA | GCTGCAAATGAGCTGACAAG |
| EED | *H. sapiens* | GGATGCTGATGCTGATGAAA | ATAGCATTTCCATGGCCAAC |
| RBBP4 | *H. sapiens* | GGCAAATGGCAGAGAACATT | TTAGAAAACACCCACGGTTTG |
| RBBP7 | *H. sapiens* | TGCGGATAAGACCGTAGCTT | GTTCTGGAGGCCCATCTTCT |
| CDKN3 | *H. sapiens* | CCAGAGGGGAACTGTCAAAA | TCCCAAGTCCTCCATAGCAG |
| COL1A1 | *H. sapiens* | ATGAGAAATCAACCGGAGGA | GACCAGGTTTTCCAGCTTCC |
| VAV1 | *H. sapiens* | GCATCTGCACACAAGGAG TG | TTCGTGAGCTCCACAATGTC |
| ARHGEF6 | *H. sapiens* | CCTGCACCTCCACTTAGACC | AGCATCCTCTTCCAGAGCAG |
| CAPN12 | *H. sapiens* | GAGCCTGATGAGGAGGATGA | AGCAGCTCCTCTGGAATCTG |
| hGAPDH | *H. sapiens* | gacctgacctgccgtctagaaaa | accaccctgttgctgtagccaaat |
| hHPRT | *H. sapiens* | GGGAGGCCATCACATTGTAG | CCTGACCAAGGAAAGCAAAG |
| mGAPDH | *M. musculus* | AACTTTGGCATTGTGGAAGG | ACACATTGGGGGTAGGAACA |
| mHPRT | *M. musculus* | AAGCTTGCTGGTGAAAAGGA | TTGCGCTCATCTTAGGCATTT |
| FOXP3 | *M. musculus* | AAACAAAGTAAGAGAGCAAAG | AGACCTCGCTCTTCTAATAATCCAA |
